# Modifying the m^6^A brain methylome by ALKBH5-mediated demethylation: a new contender for synaptic tagging

**DOI:** 10.1038/s41380-021-01282-z

**Published:** 2021-10-19

**Authors:** Braulio Martinez De La Cruz, Robert Markus, Sunir Malla, Maria Isabel Haig, Chris Gell, Fei Sang, Eleanor Bellows, Mahmoud Awad Sherif, Denise McLean, Anbarasu Lourdusamy, Tim Self, Zsuzsanna Bodi, Stuart Smith, Michael Fay, Ian A. Macdonald, Rupert Fray, Helen Miranda Knight

**Affiliations:** 1 Division of Cells, Organisms and Molecular Genetics, School of Life Sciences, University of Nottingham, Nottingham, UK; 2 School of Life Sciences Imaging Facility, University of Nottingham, Nottingham, UK; 3 Deep Seq: Next Generation Sequencing Facility, University of Nottingham, Nottingham, UK; 4 Children’s Brain Tumour Research Centre, School of Medicine, University of Nottingham, Nottingham, UK; 5 Division of Plant Sciences, School of Biosciences, University of Nottingham, Nottingham, UK; 6 Nanoscale and Microscale Research Centre, University of Nottingham, Nottingham, UK; 7 Division of Physiology, Pharmacology and Neuroscience, School of Life Sciences, University of Nottingham, Nottingham, UK; 8 Present Address: MRC Laboratory of Molecular Cell Biology, UCL, London, UK

**Keywords:** Neuroscience, Genetics, Cell biology, Psychiatric disorders, Molecular biology

## Abstract

Synaptic plasticity processes, which underlie learning and memory formation, require RNA to be translated local to synapses. The synaptic tagging hypothesis has previously been proposed to explain how mRNAs are available at specific activated synapses. However how RNA is regulated, and which transcripts are silenced or processed as part of the tagging process is still unknown. Modification of RNA by N6-methyladenosine (m^6^A/m) influences the cellular fate of mRNA. Here, by advanced microscopy, we showed that m^6^A demethylation by the eraser protein ALKBH5 occurs at active synaptic ribosomes and at synapses during short term plasticity. We demonstrated that at activated glutamatergic post-synaptic sites, both the YTHDF1 and YTHDF3 reader and the ALKBH5 eraser proteins increase in co-localisation to m^6^A-modified RNAs; but only the readers showed high co-localisation to modified RNAs during late-stage plasticity. The YTHDF1 and YTHFDF3 readers also exhibited differential roles during synaptic maturation suggesting that temporal and subcellular abundance may determine specific function. m^6^A-sequencing of human parahippocampus brain tissue revealed distinct white and grey matter m^6^A methylome profiles indicating that cellular context is a fundamental factor dictating regulated pathways. However, in both neuronal and glial cell-rich tissue, m^6^A effector proteins are themselves modified and m^6^A epitranscriptional and posttranslational modification processes coregulate protein cascades. We hypothesise that the availability m^6^A effector protein machinery in conjunction with RNA modification, may be important in the formation of condensed synaptic nanodomain assemblies through liquid-liquid phase separation. Our findings support that m^6^A demethylation by ALKBH5 is an intrinsic component of the synaptic tagging hypothesis and a molecular switch which leads to alterations in the RNA methylome, synaptic dysfunction and potentially reversible disease states.

## Introduction

In eukaryotes, N6-methyladenosine (m^6^A) and the cap-adjacent N6,2′‐O‐dimethyladenosine (m^6^A_m_), are highly abundant and reversible mRNA modifications [1, 2]. m^6^A/m^6^Am modification is regulated by effector proteins (writers, erasers and readers) which orchestrate the transfer of methyl groups to and from adenosines located internally or at the first transcribed position. Demethylase eraser effector proteins remove m^6^A modifications and include FTO and ALKBH5. Whilst the best characterised m^6^A-binding proteins (readers), which recognise specific chemical modifications and direct binding events [3, 4], belong to the YTH domain family of proteins [5–7]. Among them, the cytoplasmic YTHDF1, YTHDF2 and YTHDF3 proteins have similar sequence identity and binding affinities toward preferred RNA motifs [8, 9] and recent studies support dosage dependent redundancy in their function to regulate m^6^A dependent mRNA stability and translation [10–12]. The mechanisms which govern this redundancy in a spatio-temporal manner, and hence define the exact role of individual YTHDF reader and eraser proteins in subcellular structures, is still largely unknown.

Synaptic plasticity is widely accepted as the main neural basis of learning and memory. Plasticity processes involve changes in synaptic strength which require mRNA transcribed in the nucleus as well as local translation of mRNA at dendritic sites [13]. Activity-dependent plasticity processes occur in two phases, with an early, short term, encoding phase (E-LTP) and a late phase occurring >4 h later (L-LTP) [14]. New protein synthesis, whether initiated through activity-dependent transcription in the nucleus or translation of pre-existing ‘stalled’ transcripts on dendritic ribosomes, contributes to these phases [13, 15]. The synaptic tagging and capture hypothesis (STC) has been proposed to explain how mRNAs are localised to, and available at, specific activated synapses [16–18]. Although the STC model was originally conceptualised as a cellular model of memory formation and established by examining LTP in hippocampal tissue, the model is relevant to other forms of plasticity and cognition [19–22]. However, what constitutes the ‘synaptic tag’ remains unclear, and it may include processes that regulate RNA stability, RNA degradation and protein translation.

We hypothesise that m^6^A methylation is integral to a ‘synaptic tagging and capture’ mechanism. Supporting this, knockout of the reader, YTHDF1 in mice, results in impaired synaptic transmission and learning and memory deficits and knockdown of YTHDF1 in mouse cultured neurons results in abnormal spine morphology and impaired synaptic transmission [23, 24]. To test our hypothesis, and using advanced microscopy, we directly investigated the spatio-temporal relationships between m^6^A-modified RNAs, and the YTHDF1 and YTHDF3 readers and the demethylase ALKBH5 within synaptic subdomains. We examined changes induced by NMDA-mediated early and late phase plasticity stages, and at actively translating synaptic ribosomes, as well as during the periods of synaptogenesis and synaptic maturation in co-cultures of differentiating neurons, astrocytes and oligodendrocytes. Furthermore, by m^6^A mapping of grey and white matter from the human parahippocampus and late stage foetal brain, we characterised m^6^A regulated processes and m^6^A methylome characteristics in neuronal and glial cell-rich hippocampal tissue and during late-stage brain development.

## Materials and methods

### Neuronal cell and neuronal stem cell differentiation and culture

To explore if and how m^6^A methylation molecular processes might contribute to the spatial and temporal changes proposed in synaptic tagging and capture mechanisms, we chose to examine the relationships between m^6^A effector machinery and modified RNAs in synaptic subdomains during plasticity phases in two cell human neuronal cell lines. The neuroblastoma cell line SH-SY5Y and cerebellar medulloblastoma cell line TE671 can be differentiated from a neuroblast-like state into mature human neuronal cells through the use of different methods e.g. the addition of retinoic acid and dibutyryl-cyclic-adenosine-monophosphate (dbcAMP) [25, 26]. When differentiated, cells extend long, branched processes and demonstrative properties of neuron subtypes such as cholinergic and glutamatergic neurons [27]. We chose to examine both differentiated cell lines to demonstrate robustness in findings. TE671 and SH-SY5Y cells were cultured under standard conditions and in complete Dulbecco’s Modified Eagle Medium (DMEM) and complete DMEM/F12 (1:1) Nutrient Mixture Medium, respectively. Cells were maintained at a passage number under ten and allowed to grow until ~90% confluency. TE671 cells were differentiated in serum-free DMEM and SH-SY5Y cells in Neurobasal Medium supplemented with 0.5 mM GlutaMAX (Thermo Fisher Scientific), B-27 supplement (2 ml per 100 ml of medium), and 1% Penicillin/Streptomycin (10,000 U/ml). The differentiator compound, dbcAMP, was added at a final concentration of 400 µM to TE671 cultures whereas retinoic acid at a final concentration of 10 μM was added to SH-SY5Y cells.

The human neural progenitor cell line, ReNcell CX, (EMD Millipore/Merck) is a neural stem cell line derived from the ventral mesencephalic region of the developing human brain that differentiates into co-cultures of functional neurons, astrocytes and oligodendrocytes. It has been used as a model of human neural development and differentiation [28]. The ReNcell CX cell line was expanded on laminin coated flasks (20 μg/ml, Sigma-Aldrich) in ReNcell NSC Maintenance Medium containing fresh EGF (20 ng/ml) and FGFb (20 ng/ml; EMD Millipore). Cultures were incubated at 37 °C in a humidified atmosphere of 5% CO_2_. ReNcell CX neural progenitor cells were differentiated into co-culture of neuronal, astrocytes and oligodendrocytes by replacing the complete ReNcell CX medium with EGF and FGFb free medium. They were subsequently fixed at three time points, 0, 1 and 14 days. Cell authentication was performed for all three cells lines examined, e,g, TE671 and SH-SY5Y and ReNcell CX, by microscopy observations of cellular morphology and growth curve analysis.

### Neuronal activation and calcium imaging and ribopuromycilation assays

To activate NMDA receptors or to depolarise voltage-gated calcium channels specifically at synapses or throughout neuronal processes, dTE671 and dSH-SY5Y cells were differentiated and 100 µM NMDA or 30 mM KCl were added to the media. To stop the process after 15 min or 24 h, the media was removed and the cells were fixed. To confirm the cellular response to NMDA or KCl, calcium imaging was performed on live differentiated cultures using a fluorescent calcium indicator Fluo4-AM (ThermoFisher) diluted in 20% pluronic acid in DMSO (ThermoFisher) and culture media for a final Fluo4-AM concentration of 100 µM. Cells were incubated at 37 °C for 45 min in the dark, followed by washes in HBSS and a 30 min incubation. Coverslips were loaded on a LSM710 confocal microscope (Carl Zeiss, Germany) and videos were captured using a C-Apochromat 40×/1.2 water objective, 488 nm laser excitation and emission between 495 and 635 nm after compound applications (Supplementary Information Video S1 and S2).

A ribopuromycilation assay was conducted to examine actively translating ribosomes and followed a protocol described by David et al. [29]. In brief, Puromycin (Sigma-Aldrich, P7255), a protein synthesis inhibitor, and Emetine (Merck Millipore, 324693), a chain elongation inhibitor, were added to a final concentration of 18.4 µM and 208 µM, in wells or plates respectively. Cells were incubated at 37°C (5% CO_2_) for 5 min and washed. Cells were immediately fixed using 4% Paraformaldehyde and permeabilised for 15 min using labelling buffer (0.05% saponin, 10 mm glycine, 5% foetal bovine serum In 1×PBS), and immunolabelling was performed using an anti-Puromycin antibody. All NMDA/KCL stimulation and ribopuromycilation experiments were repeated twice in each condition.

### Immunocytochemistry

Cells were fixed using 4% Paraformaldehyde and permeabilized with 0.2% Triton X-100 and incubated with primary antibodies for two hours. After washing with 1xPBS, cells were incubated with Alexa Fluor-conjugated secondary antibodies for one hour. Coverslips with immunostained cells were mounted using Fluoroshield DAPI (Sigma-Aldrich, F6057) medium and sealed. A full list of primary and secondary antibodies and the concentrations used are presented below. Several primary antibodies specific for m^6^A/m^6^Am modifications were assessed and included mouse monoclonal anti-m^6^A (Merckmillipore, MABE 1006, clone 17-3-4-1), rabbit monoclonal anti-m^6^A (Abcam, ab190886), and rabbit anti-m^6^A New England Biolabs (NEB E1610S, USA). These antibodies were compared using confocal microscopy and indicated a 1.00 correlation (Mander’s M1 and M2 coefficients) in immunoreactivity supporting that they bind to the same entity (Supplementary Fig. S1).

### Advanced microscopy

Cells were visualised using both a confocal LSM880 microscope (Carl Zeiss, Germany) and super resolution Zeiss ELYRA PS.1 microscope (Carl Zeiss, Germany). Confocal images were captured at a 12-bit depth and with consistent settings between samples using a 63× Plan-Apo oil objective (NA = 1.4). The green channel was excited at 488 nm and emission recorded at 520 nm. The red channel was excited at 561 nm and emission recorded at 605 nm. The far red channel was excited at 633 nm and emission recorded at 670 nm. All channels had an emission recording bandwidth of 40 nm. Z-stacks were collected at a spacing of 1 µm. At least 20 images per condition were recorded from two cover slips and on two separate days, generating approximately ~800 activation and ~100 ribopuromycilation confocal images. Negative controls without a primary antibody were captured for each condition.

For super resolution microscopy, cells were differentiated on square high-precision 22 × 22 mm coverslips (Zeiss, 474030-9020-000) and post-fixed for 10 min in 4% PFA and washed in PBS before mounting using CFM3 non-hardening medium (CitiFluor, Pennsylvania, CFM3-25). Slides were kept at room temperature and imaged at 27 °C using a Plan Apochromat 63×/1.4 oil DIC M27 objective with Zeiss Immersol™ 518F (30 °C) oil (Zeiss, 444960-0000-000). In a stack scan mode, two tracks were setup for lasers 488 nm and 561 nm at 25% power and with 100 ms exposure times. SRM SIM grating periods were 28.0 µm and 34.0 µm for green and red tracks, respectively. Bandpass (BP) filters used were BP420–480 + BP 495–550 + LP 650 for green and BP 420–480 + BP 570–640 + LP 740 for red. Multiple Z-stacks were captured for all experiments. Channel shift correction was performed using both 100 nm TetraSpeck Microspheres (ThermoFisher Scientific, T7279) and marker-based alignment in Zen Black 2012 software (Carl Zeiss, Germany). Quantification of colocalization in SRM SIM images was not performed owing to rapid bleaching of the fluorescent antibodies. Colocalization analysis and 3D modelling and movies were generated using the Zen Black colocalisation and 3D modules. At least ten images were captured per condition for each repeat in the NMDA and KCL activation and ribopuromycilation experiments generating approximately ~800 activation and ~100 ribopuromycilation confocal images.

Quantitative localisation at synapses was performed on confocal images using Fiji software and an in-house methodology which followed standard imaging analysis principles outlined in [30]. A three channel approach was used to select synapse-specific regions. The third channel, the synaptic marker, was manually thresholded to eliminate background and converted to an 8-bit mask image to create regions of interest. Using the Coloc2 built-in colocalisation analysis tool, images were autothresholded and colocalisation between the remaining two channels was evaluated at the synaptic regions of interest. For presentation purposes, a gamma correction of 0.45 was applied to the selected representative images. All experiments were repeated twice and at least five images were captured in each condition. Pearson’s Correlation Coefficients were calculated using, and statistical analysis performed in, GraphPad Prism. A two-way ANOVA (two sided) was performed to compare the interaction and difference in colocalisation of m^6^A and related proteins at post-synaptic regions in activated cells by the two compounds and over two time points (15 min and 24 h). An unpaired *t* test was performed to compare differences in colocalisation after activation at active ribosomes and m^6^A-binding proteins at synaptic areas. A one-way ANOVA (two sided) was performed to compare differences in m^6^A abundance in stem cells differentiating over time. Statistical significance was considered at *p* < 0.05.

### Scanning transmission electron microscopy (STEM)

Mouse (C57/BL6 strain) hippocampal tissue was fixed with 3% PFA/0.1% Glutaraldehyde in 0.1 M phosphate buffer through perfusion methods and polymerised in araldite. 200 nm sections were mounted on graphene oxide grids. Immunolabelling was performed overnight using the anti-m^6^A (Abcam, ab190886) or anti-YTHDF1 (Abcam, ab99080) antibodies at 1:25 concentrations. After washing, the grids were incubated with 5 nm gold nanoparticle-conjugated secondary antibody for 4 h (Sigma-Aldrich, G7277). Scanning TEM images were obtained on a JEOL 2100F TEM operating at 200 kV, equipped with JEOL bright field and dark field detectors. STEM tilting involved a tilt series acquisition at five degree steps. Images were acquired and processed using Gatan Digital Micrograph software and the open source Fiji software [31]. By use of the diffuse, high angle annular diffracted electrons in STEM, the contrast mechanism for the subsequent image is significantly proportional to the atomic number Z ^ 1.6–1.9 per atom, increasing the contrast between gold and organic molecules [32].

### m^6^A-sequencing

Non-diseased grey matter and white matter tissue from the parahippocampal region were obtained from the Children’s Cancer and Leukaemia Group (CCLG) through the Nottingham Children’s Brain Tumour Research Centre. Consent and ethical approval was obtained by CCLG (Ethics Committee approval, 06/MRE04/86). Foetal brain RNA post conception weeks (PCW) 22–30 was acquired from clontech #636526. 40–50 mg of snap frozen tissue was used for RNA extraction using the mirVanaTM miRNA Isolation kit (Applied Biosystems, Carlsbad, CA, USA). RNA was treated with DNase (Promega, 2 U). Total RNA was measured using Qubit RNA BR assay kit (Life technologies, Q10210). RNA QC was performed using Agilent Bioanalyser RNA Nano 6000 kit (Agilent biotechnologies, 5067-1511). 2 µg of Total RNA was used for rRNA depletion using Ribozero gold rRNA removal kit (Illumina, MRZG12324). Ribo-depleted RNA was fragmented to 100 bp using NebNext Fragmentation module (NEB, E6150S). 1/10 volume of fragmented RNA was separated to use as input control. 5 μg of m^6^A Ab (abcam, ab151230) was added to 25 μl of protein G beads (NEB, S1430) in 250 μl of IPP buffer and incubated at 4 °C. Following incubation, beads were washed and RNA eluted in RLT buffer (Qiagen, 79216) and purified using 20 μl of MyOne Silane Dynabeads (Life Technologies, 37002D).

First and second strand synthesis was performed using the NEBNext RNA First strand synthesis module (NEB, E7525S) and Ultra-Directional RNA Second Strand Synthesis Module (NEB, E7550S) respectively. Sequencing libraries from double stranded cDNA were generated using NEBNext^®^ Ultra II DNA Library Prep Kit for Illumina (NEB, E7645S) according to manufacturer’s instructions. Library QC was performed using bioanalyser HS kit (Agilent biotechnologies, 5067-4626). Libraries were quantified using qPCR (Kapa Biosystems, KK4824) and sequenced according to manufacturer’s instructions using Illumina NextSeq500 platform to generate ~40–50 million 75 bp PE reads per sample (Illumina, FC-404-2002).

Preprocessing of reads was performed by FASTQC (version 0.11.5). Adaptor sequences were removed and trimming was performed with Scythe/Sickle toolkits. Reads were aligned to the human genome (build GRCh37/hg19) using HISAT2 (version 2.0.5) [33] with default settings. Stringtie (version 1.3.3) [34] was used to calculate read coverage, Fragments Per Kilobase Million, and Transcripts Per Kilobase Million from uniquely mapped reads. N6-methyladenosine peaks were identified using the exomePeak calling algorithm [35] using default settings and with the input library as background.

Modified transcripts and regions were annotated to eight non-overlapping transcript segments using HOMER (Salk Institute, USA) [36, 37]. These segments were: intron, exon, transcription termination site (TTS ± 100 bp from stop site), transcription start site (TSS ± 100 bp from start site), 3′ UTR, 5′ UTR, non-coding, and intergenic. m^6^A motifs were identified using DREME [38]. Transcripts were examined for multi-modification (*N* > 3), i.e. the number of district m^6^A peaks detected, and included only distinct m^6^A peaks with distances larger than 400 bps along the transcript. Functional gene ontology annotation was performed using DAVID’s Functional Annotation Clustering [39] and assessed all modified transcripts as well as sub groups of transcripts with modifications identified at specific regions, e.g. 3′ UTR, 5′ UTR, non-coding, and intergenic.

### Antibodies

The following primary antibodies and dilutions were used: mouse anti-m^6^A (Merck Millipore, MABE 1006, clone 17-3-4-1) 1:250, mouse anti-YTHDF3 (Santa Cruz Biotech, sc-377119) 1:100, and mouse anti-Puromycin (Merck Millipore, MABE343) at 1:10000, rabbit anti-Dcp1a (Abcam, ab47811) 1:250, rabbit anti-m^6^A (Abcam, ab190886) 1:250, rabbit anti-VGluT1 (Abcam, ab72311) 1:500, rabbit anti-DLG4 (Sigma-Aldrich, HPA010122) 1:250, rabbit anti-YTHDF1 (Abcam, ab99080) 1:100, rabbit anti-ALKBH5 (Merck Millipore, ABE1013) 1:100, rabbit anti-L7A (Cell Signalling Technology, R225) 1:250, rabbit anti-FMR1 (Sigma-Aldrich, HPA050118) 1:250, goat anti-VGluT1 (Abcam, ab110139) 1:100, and goat anti-PSD95 (Abcam, ab12093) 1:100. The secondary antibodies were donkey anti-mouse AF488 (Abcam, ab150105) 1:500, goat anti-rabbit AF568 (Abcam, ab175471) 1:500, and donkey anti-goat AF647 (Sigma-Aldrich, SAB4600175).

## Results and discussion

### Advanced microscopy spatial mapping of m^6^A complexes at synapses

Using antibodies specific for m^6^A modification, we used confocal microscopy to characterise m^6^A abundance and distribution in two differentiated human neuronal cell lines (dSH-SY5Y and dTE671). We observed m^6^A modified transcripts throughout all cytoplasmic regions, including axons and dendrites, but an m^6^A signal was absent from the nucleus. Double labelling of m^6^A and VGluT1, a marker of presynaptic terminals, and PSD-95/DLG4, a marker of postsynaptic sites, provided evidence of co-localisation (Supplementary information file, Fig. S2), indicating that m^6^A tagged RNAs are at both the pre- and post- synaptic terminals.

**Fig. 1:**
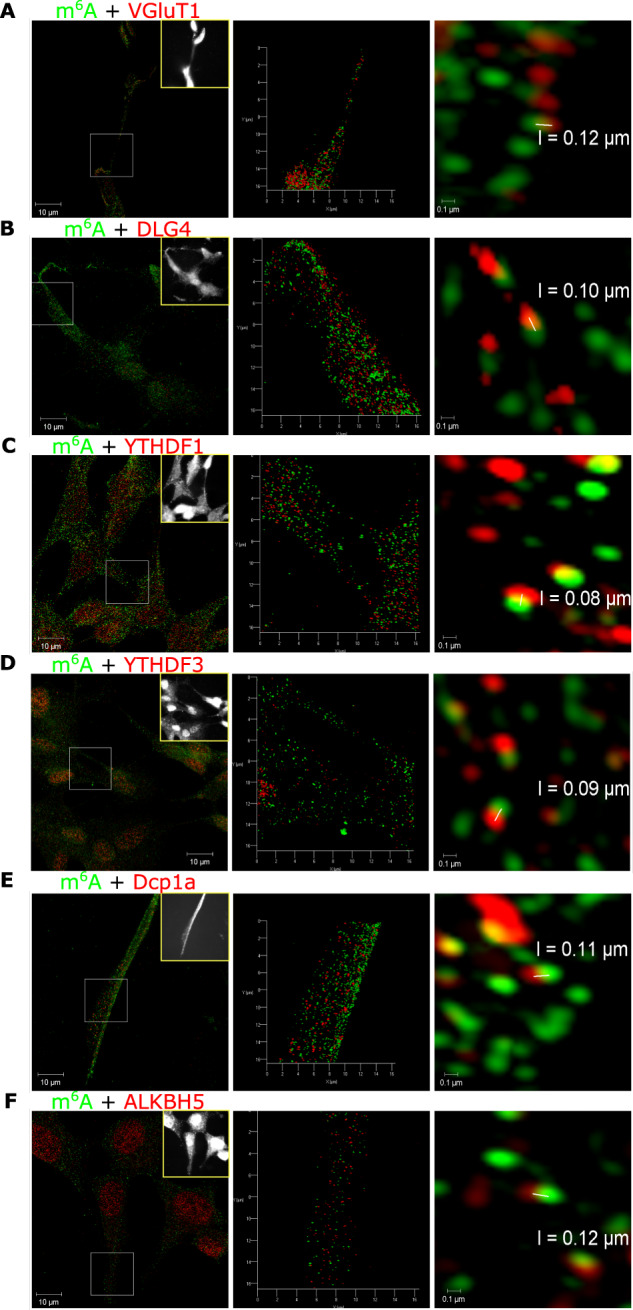
Spatial mapping of m^6^A-modified transcripts and m^6^A-binding proteins at synapses. Super resolution structured illumination 3D microscopy of differentiated human neuronal cells shows colocalisation of m^6^A modified transcripts (green) with **A** presynaptic VGluT1 (red), **B** post synaptic PSD-95/DLG4 (red), **C** YTHDF1 reader (red), **D** YTHDF3 reader (red), **E** Dcp1a (red), **F** ALKBH5 eraser (red). Left column, maximum intensity projections SIM images of the widefield grey scale inset image (yellow box). Middle column, 3D models of zoomed in regions of interest (white squares in the left column). Right column, resolution at which two fluorescent puncta can be discerned (range 80–120 nm).

To examine m^6^A modified transcripts within the cytoplasm and at synaptic sites at a higher resolution (i.e. 80–120 nm), we visualised cells using super-resolution Structured Illumination Microscopy (SIM). We observed m^6^A modified transcripts within 1 µm of VGluT1 or DLG4/PSD-95 (Fig. 1 A–B, video). We also observed a moderate abundance of overlap in fluorescence signals for m^6^A modified transcripts and m^6^A readers, i.e. YTHDF1 (~13%) and YTHDF3 (~28%), confirming that modified transcripts are in near proximity to both reader proteins in the cytoplasm (Fig. 1 C and D, Supplementary information video S3). However, Dcp1a, a marker of RNA processing bodies (P bodies) and mRNA decay sites, showed only a few occurrences of overlap with m^6^A modified transcripts (Fig. 1E). Similarly, ALKBH5, an eraser protein, was found to be mostly located in the nucleus (Fig. 1F). However, we observed some ALKBH5 colocalisation with m^6^A modified RNA within the cytoplasm suggesting that in addition to nuclear RNA mechanisms, e.g. nuclear RNA export, splicing [40, 41], m^6^A demethylation has a function in RNA processing outside the nucleus.

Upon image rotation of the SIM 3D images, some instances of apparent colocalisation between m^6^A and synaptic markers were found to have gaps of approximately 120 nm (Supplementary information file, Fig. S3 A, B). Image rotation also indicated that the m^6^A readers YTHDF1, YTHDF3, eraser ALKBH5 as well as the P body marker Dcp1a, were always adjoining to m^6^A-modified transcripts (Supplementary information file, Fig. S3, C–F). Moreover, Dcp1a was commonly adjacent to two m^6^A modification signals suggesting that there is clustering of Dcp1a with modified transcripts. These results indicated that m^6^A-mediated regulation of protein expression is important at pre- and postsynaptic sites, and that in differentiated but quiescent, i.e. non-synaptically activated cells, m^6^A modified RNA is bound to the reader proteins, YTHDF1 and YTHDF3 more often than to the eraser ALKBH5.

### m^6^A dynamics are plasticity-time dependent and involves ALKBH5 translocating out of the nucleus

**Fig. 2:**
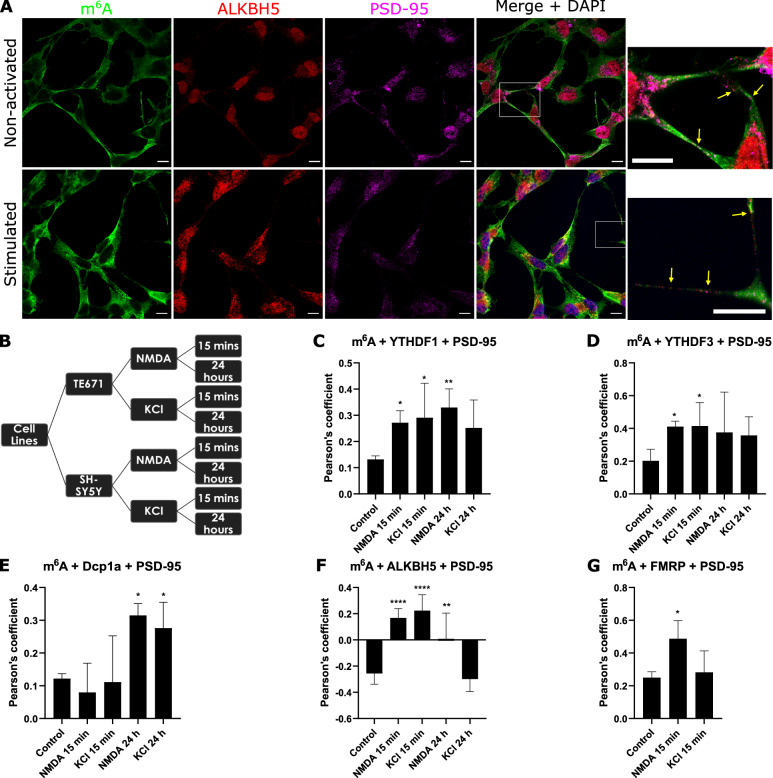
m^6^A regulation at synapses is activity-time dependent and involves m^6^A demethylation. RNA binding proteins change in proximity to modified transcripts after synaptic stimulation as imaged by confocal microscopy. **A** 15 min after NMDA activation of dSH-SY5Y cells induces increased colocalisation between m^6^A-RNAs (green), the eraser protein ALKBH5 (red) and post synaptic marker, PSD-95 (magenta). Insets of selected regions are shown on the right. **B** Experimental design. Mean Pearson’s Correlation Coefficient for m^6^A colocalisation with YTHDF1 (**C**), YTHDF3 (**D**), Dcp1A (**E**), ALKBH5 (**F**) and FMRP (**G**) at postsynaptic sites at 15 min or 24 h after NMDA and KCl application in dSH-SY5Y cells. Error bars denote 95% CI. *p ≤ 0.05, **p ≤ 0.005, ***p ≤ 0.0005, ****p ≤ 0.00005.

As m^6^A modified transcripts and m^6^A-binding reader proteins are abundant within neuronal processes, we performed triple-labelling immunofluorescence and confocal microscopy to assess m^6^A modified mRNAs and m^6^A-binding proteins directly at synapse-specific regions of interest. (Fig. 2A, top row; Supplementary information file, Fig. S4) in dTE671 and dSH-SY5Y cells. In differentiated but non-synaptically stimulated cells, we observed modest colocalisation (Pearson’s Correlation Coefficients [PCC] values of ~0.20) between m^6^A modified transcripts and YTHDF1/YTHDF3/Dcp1a at pre- and post- synaptic ROIs (Fig. 2C–E and Supplementary information file, S4). In contrast, ALKBH5 displayed a negative correlation at post synaptic sites (dSH-SY5Y PCC −0.25 SEM 0.02; dTE671 PCC −0.23, SEM 0.02) again suggesting that during quiescence the eraser protein is not commonly in the vicinity of modified transcripts (Fig. 2F and Supplementary information file S4).

We next examined spatial-temporal patterns of m^6^A-modified transcripts and m^6^A effector proteins at synapses after activation of NMDA receptors or cell membrane depolarisation by voltage-gated calcium channels. A cellular response to NMDA or KCl was confirmed by calcium imaging (Supplementary information videos S1-2). We examined the responses to stimulation at two different time points, 15 min or 24 h after application, which are analogous to early E-LTP and late L-LTP phases. We found significant differences in co-localisation of m^6^A mRNAs and m^6^A-interacting proteins before and after synaptic stimulation at pre- and post- synaptic regions (Fig. 2 and Supplementary information file Fig. S4). At postsynaptic sites, colocalisation of m^6^A modified transcripts with the readers YTHDF1 and YTHDF3 increased significantly at 15 min after application of NMDA or KCl in dSH-SY5Y (*p* < 0.05) and dTE671 (*p* < 0.005) cells and remained high at 24 h (Fig. 2C, D, Supplementary information file S5) indicating the involvement of both these m^6^A reader proteins in regulating local RNA processing during postsynaptic plasticity processes. Conversely, Dcp1a, a marker of RNA decay sites, was significantly increased (*p* < 0.05) only at 24 h after NMDA or KCl application (Fig. 2E) suggesting that some modified transcripts are degraded during a late phase of LTP.

In contrast to a non-stimulated state, when the ALKBH5 eraser protein showed a negative correlation with m^6^A abundance, 15 min following NMDA application there was a significant increase (*p* < 0.00005) in ALKBH5 abundance and m^6^A modified mRNA colocalisation at postsynaptic sites (dSH-SY5Y PCC 0.17 SEM 0.05; dTE671 PCC 0.17, SEM 0.02) (Fig. 2F). This suggests that the eraser protein is transported from the nucleus out to cytoplasmic synaptic sites during early plasticity as evident in Fig. 2A. At presynaptic sites, colocalisation of m^6^A modified RNAs with YTHDF3 and ALKBH5 was also significantly increased at 15 min after NMDA or KCl stimulation (*p* < 0.005) but only YTHDF3 remained increased at 24 h in dTE671 cells, *p* < 0.05 (Supplementary information file, Fig. S5). Together, these results demonstrate that the reader effector proteins are involved in RNA regulation during both short term and longer term plasticity phases at both pre- and post- synaptic sites after NMDA activation and that ‘demethylation’ by the ALKBH5 eraser protein is part of the mechanism following short term synaptic plasticity.

The Fragile X Mental Retardation Protein (FMR1/FMRP), which causes the neurodevelopmental delay disorder fragile X syndrome, is reported to transport transcripts to post-synaptic sites following NMDA receptor activation and to repress translation by stalling mRNA-linked ribosomes [42, 43]. To gain insight into the involvement of FMRP in m^6^A mechanisms, we assessed whether FMRP is in close vicinity to m^6^A modified RNAs at synapses, and whether stimulating with NMDA or KCl changes this relationship. We found that colocalisation in non-stimulated cells was modest (PCC of 0.25) at postsynaptic sites. After activation with NMDA, FMRP colocalisation with m^6^A-methylated transcripts at postsynaptic sites significantly increased (*p* < 0.05, dSH-SY5Y; *p* < 0.005, dTE671 Fig. 2G). However, applying KCl did not cause a significant change in FMRP localisation with modified transcripts suggesting perhaps that a local rise of [Ca^2+^]_i_ near synapses is needed for the association of FMRP. These results provide evidence that FMRP is involved in m^6^A-regulatory mechanisms influencing short term plasticity at glutamatergic synapses.

### Demethylation at local active ribosomes

**Fig. 3:**
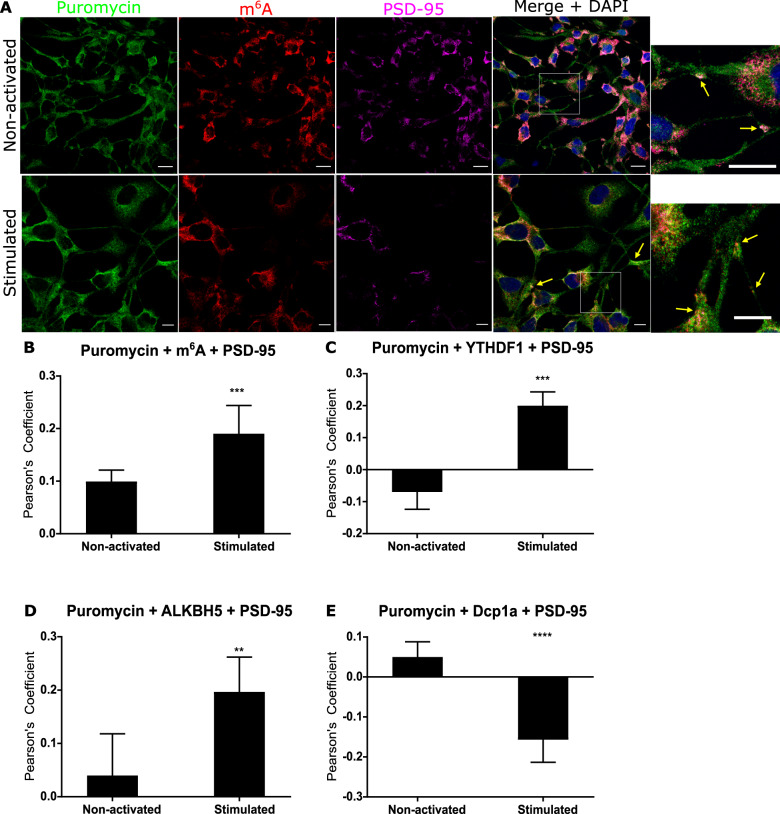
m^6^A-modified RNAs increase at actively translating synaptic ribosomes 15 min after synaptic activation. **A** Confocal microscopy indicates non-activated and NMDA activated dTE671 cells show regions of colocalisation between m^6^A-modified RNA (red) at post synaptic sites (PSD-95, magenta) and translationally active ribosomes indicated by puromycin binding (green). Mean Pearson’s Correlation Coefficient calculated for colocalisation between actively translating ribosomes 15 min after activation and (**B**) m^6^A modified transcripts (**C**) YTHDF1 reader, (**D**), ALKBH5 eraser (**E**) and Dcp1a in postsynaptic areas. Error bars denote 95% CI. *p ≤ 0.05, **p ≤ 0.005, ***p ≤ 0.0005.

Given that the increased abundance of m^6^A**-**modified transcripts colocalised with reader and eraser proteins after short term glutamatergic activation, we sought to confirm that this process was occurring at actively translating ribosomes local to synapses. Using protein synthesis inhibitors and a ribopuromycilation immunofluorescence assay, we stalled translation in dTE671 cells before and 15 min after NMDA receptor activation and quantified actively translating ribosomes with m^6^A modified RNAs and YTHDF1, YTHDF3 and ALKBH5 at post-synaptic synapses. We found that there was no difference between the quiescent and stimulated state in the amount of fluorescence at active ribosomes throughout the whole cell [unpaired *t* test, *p* = 0.48] (Supplementary information Fig. S6). Furthermore, although Dcp1a was found abundant at synapses before synaptic stimulation, the Dcp1a protein showed low colocalization with translating ribosomes, and after exposure to NMDA co-localisation was further reduced (PCC −0.157, *p* < 0.005) (Fig. 3E). These data are consistent with a lack of mRNA degradation during local synaptic protein synthesis.

In contrast, there was a significant increase in m^6^A modified transcripts (*p* < 0.0005) (Fig. 3B) and increased abundance of YTHDF1 (*p* < 0.0005) and ALKBH5 (*p* < 0.005) after synaptic activation at active ribosomes at postsynaptic sites (Fig. 3A, C, D, Supplementary information Fig. S7). These findings strongly suggest that during the early phase of NMDA mediated plasticity in post synaptic compartments, the RNA reader proteins along with modified transcripts are involved in RNA translation. The significant increase in colocalisation of ALKBH5 with actively translating polyribosomes, also provides evidence for a role of demethylation in local protein synthesis during early plasticity. However, the mechanistic consequences of de-modification and involvement in translational processing, i.e. during the different stages of translation, the timing and the targeting of specific modified populations, still remain to be determined and confirmed.

### m^6^A-RNAs increased abundance with synaptic maturation

**Fig. 4:**
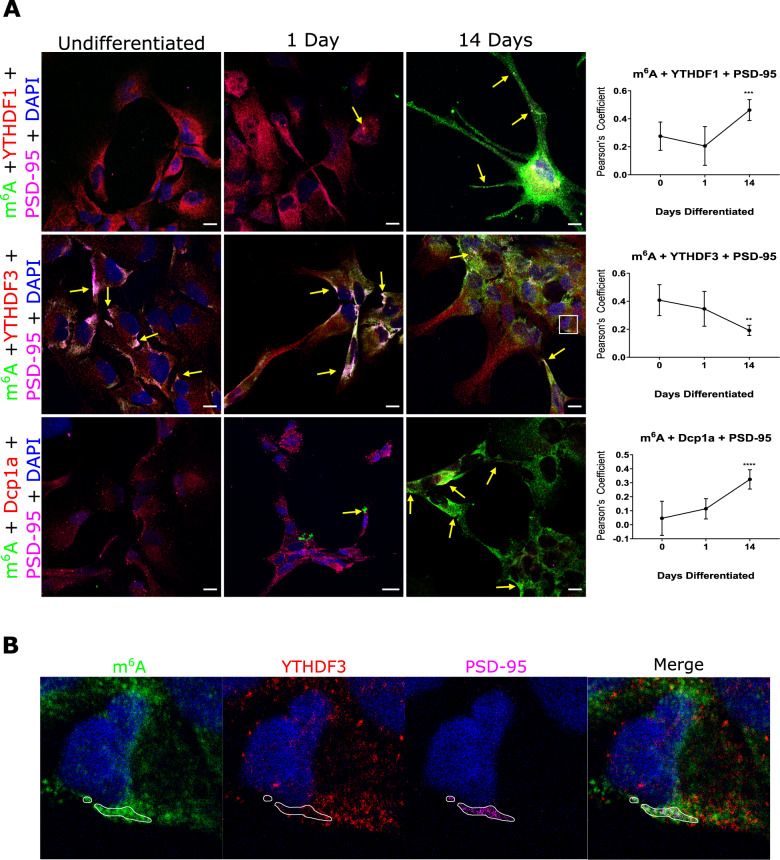
m^6^A modified RNAs increase, and colocalization with YTHDF3 decreases, at synapses during synaptic maturation. **A** Human neural progenitor cells examined by confocal microscopy show m^6^A-modified RNAs (green) increase in abundance during synaptic maturation (1 and 14 days differentiation) and increase in co-localisation at postsynaptic sites (magenta) with YTHDF1 (red) and Dcp1a (red) but decreases with YTHDF3 (red). Arrows point to regions where there is high white fluorescence signal indicating high colonisation of modified RNA and effector/dcp1a proteins at synapses. Far right, Pearson’s Correlation Coefficients for colocalisation between m^6^A modified transcripts, m^6^A reader binding proteins, YTHDF1, YTHDF3 and Dcp1a, over the three time points. **B** High magnification images showing less co-localisation between YTHDF3 (red) and m^6^A modified RNAs (green) at postsynaptic regions (PSD-95, magenta) encircled in white at day 14. Error bars denote 95% CI. *p ≤ 0.05, **p ≤ 0.005, ***p ≤ 0.0005.

m^6^A methylation has previously been associated with proliferation and differentiation in embryonic stem cells [44–46] and post-natal development of the mammalian cerebellum [47]. However, the role of specific YTH reader proteins during synaptogenesis is not known. We investigated the abundance of m^6^A modified transcripts and proximity with effector proteins at synapses during differentiation of human neuronal progenitor stem cells (hNPSCs) into co-cultures of neurons, astrocytes and oligodendrocytes. Three time points were assessed: before differentiation; one day after differentiation (early differentiated); and 14 days after differentiation (fully differentiated) – a time point which reflects late stage synaptic maturation [48]. m^6^A modified transcripts were found to be very low throughout the cytoplasm in undifferentiated and early differentiated cells (Fig. 4A). However, in fully differentiated cells, m^6^A-modified RNAs increased dramatically. Quantifying colocalisation of modified transcripts with YTH readers and the Dcp1a protein at postsynaptic sites demonstrated a significant increase in colocalisation between m^6^A-modified RNAs and YTHDF1 (*p* < 0.0005) as well as with Dcp1a (*p* < 0.0001) at 14 days, but a significant decrease in colocalisation between m^6^A and YTHDF3 (*p* < 0.005) in fully differentiated cells (Fig. 4A). Indeed, although at 14 days we observed abundant modified RNAs at synapses, YTHDF3 appeared more dispersed within the cytoplasm and appeared excluded at postsynaptic sites (Fig. 4B). These findings suggest that the influence of m^6^A regulation on synaptic differentiation processes involves both YTHDF1 at YTHDF3 effector proteins at early stages but more prominently YTHDF1 at the later stage of synaptic maturation.

To explore whether the difference in the readers’ protein localisation during synapse formation related to changes in gene expression during development, we examined RNA-seq data for 16 brain regions over post conception weeks (pcw) 4–37 and post birth years 0–40 generated by the BrainSpan Atlas of the Developing Human Brain [45]. We observed that *YTHDF1* and *YTHDF3* show clear differences in time and brain tissue specific expression patterns, and in particular, during the periods critical for synapse formation and synaptic pruning (pcw 20–37) (Supplementary Information Fig. 8). In contrast, and although the eraser *ALKBH5* has an overall higher abundance than *YTHDF1, AlKBH5* and *YTHDF1* show similar spatio-temporal patterns of expression including the synapse formation period and up to mid adulthood. These data suggest that the role of these effector proteins upon protein synthesis at synapses during the process of synaptic maturation, may be driven in part by a coordinated pattern of differential gene expression and abundance of YTHDF1 and YTHDF3.

One proposed explanation for such a switch in gene expression and co-localisation at synapses involves regulation by the antisense *YTHDF3-AS1* Lnc RNA which is located adjacent to the *YTFDH3* gene locus. *YTHDF3-AS1* transcripts are reported to interact with proteins involved in RNA processing including YTHDF2 [49, 50]; with proteins involved in hnRNP complexes [51]; and with RNA-binding proteins involved in cytoplasmic transport [52]. Furthermore, from m^6^A-sequencing data we observed that *YTHDF3-AS1*, as well as all m^6^A effector protein transcripts (Fig. 6 E), are themselves m^6^A modified in brain tissue. Thus, it may be that YTHDF1 and YTHDF3 abundance at subcellular locations is controlled by feedback systems involving m^6^A autoregulation of effector proteins, the *YTHDF3-AS1* transcript and proteins which influence RNA processing, packaging and transport. Future studies which aim to explore m^6^A effector proteins and RNA-protein interaction networks using targeted functional experimental designs, i,e genotype specific mutants or CRISPR-cas assays, would provide further insight into differential colocalization and expression.

### In situ CA3 synaptic mapping of m^6^A-RNAs

**Fig. 5:**
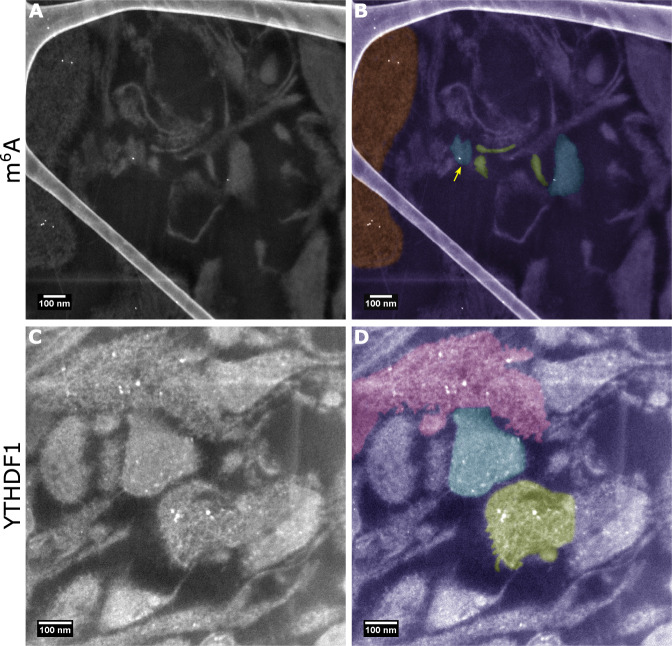
Modified RNAs are present in situ at pre- and post- synaptic sites within CA3/CA4 neurons. **A**, **B** STEM imaging indicates m^6^A modified RNAs at both at the postsynaptic compartment (coloured blue and shown by the yellow arrow), presynaptic terminals (coloured green) and in processes (orange). **C**, **D** YTHDF1 was found clustered within the presynaptic region (green) whilst in the postsynaptic sites (blue) the protein is more distributed and located at both postsynaptic terminals near to the active zone as well as throughout the bouton.YTHDF1 was also abundant within dendritic spines (pink). The structure evident on the left hand side of **A** and **B** is the amorphous carbon support film.

To examine the spatial locality of modified transcripts at the nano resolution within single mature synapses in situ, we performed scanning transmission electron microscopy imaging on primary mouse tissue from the Cornu Ammonis regions of the hippocampus. In CA3/CA4 regions, we observed modified transcripts near to presynaptic terminals and at postsynaptic densities (Fig. 5A, B) as well as in cytoplasmic processes. In addition, the YTHDF1 reader was found to be present in clusters at presynaptic sites, but had a more dispersed distribution at the postsynaptic sites including at postsynaptic terminals (Fig. 5C, D), and had a wider distribution within dendritic spines (pink region in Fig. 5D). These observations provide further evidence that m^6^A regulatory mechanisms maybe important for both pre- and post- synaptic processing within critical pathways such as the perforant pathway in the hippocampus.

### m^6^A methylome in the human parahippocampus

**Fig. 6:**
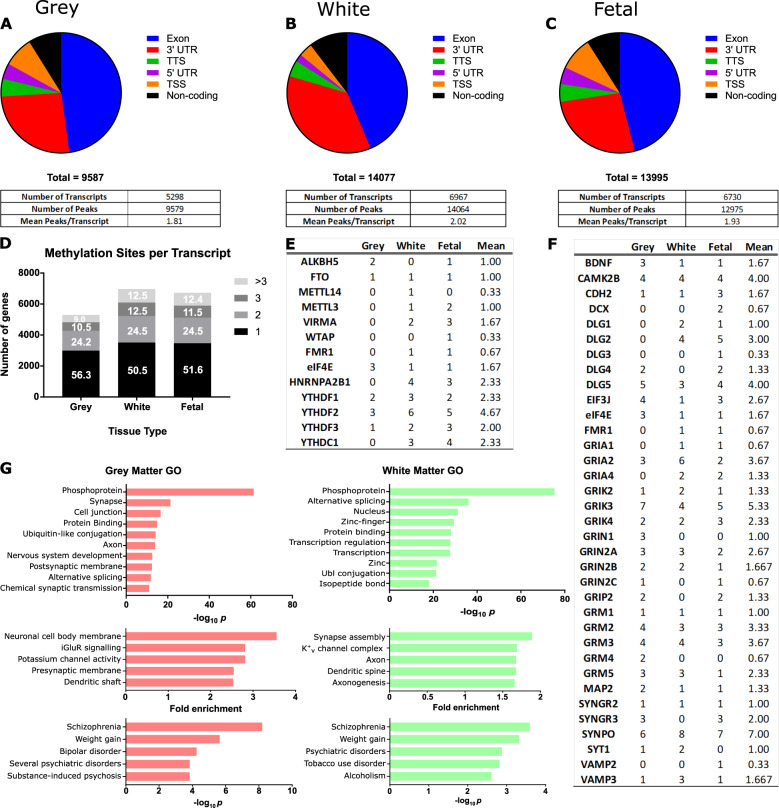
m^6^A methylome in human parahippocampus grey and white matter. Topological distribution of m^6^A sites along transcripts and number of m^6^A peaks and modified transcripts in **A** grey matter, **B** white matter and **C** foetal brain. **D** Number of m^6^A modification sites per transcript. **E** m^6^A reader, writer and eraser protein transcripts and **F** synapse related protein transcripts show multi-modification. **G** Gene ontology processes and disease terms enriched for m^6^A modified transcripts presented by most significant *P* values and largest fold enrichment in grey and white matter tissue.

The parahippocampus is composed of white matter tissue, predominately comprised of myelinated axons and glia cells such as oligodendrocytes; and grey matter, composed of cell bodies, dendrites and synapses. Although traditionally the focus of plasticity research has been on grey matter neuronal and synaptic sites, the importance of white matter myelination in plasticity mechanisms such as memory consolidation, has gained prominence [53–55]. To assess m^6^A methylation patterns across the length of individual transcripts and to characterise modified transcripts belonging to function categories within the parahippocampus, we performed high throughput m^6^A-seq of human parahippocampal grey and white matter tissue. In addition, we sequenced foetal brain tissue post conception weeks 20–33, a late development stage in which populations of neuronal and glial cells, and functional synapses, exist, to compare characteristics with the adult white and grey matter hippocampal methylome. We identified 9,579 high confidence peaks in 5298 coding transcripts in grey matter tissue; 14,064 peaks in 6968 coding transcripts in white matter tissue and 14,249 peaks in 6730 coding transcripts in foetal brain (Fig. 6 A–C). We also identified 851, 1460 and 1262 non-coding mRNAs in grey matter, white matter, foetal brain tissue, respectively. The most enriched m^6^A binding motif was found to be GGAC, consistent with the previously reported RRACH motif [56].

We found m^6^A methylation occurred predominantly within exons (~45%) and the 3′UTR (~30%) and this did not vary between tissues (Fig. 6 A–C). The mean number of methylation peaks/sites per transcript was approximately two, with a 50–25–25 percent divide in transcripts with one, two or three or more distinct m^6^A modified regions (Fig. 6D). Transcripts encoding the majority of known m^6^A writer, reader and eraser and other binding proteins were themselves modified, providing evidence for m^6^A autoregulation (Fig. 6E). We observed that the eraser, writer and FMRP protein transcripts had on average only one modified region *per* transcript. In contrast, m^6^A readers were found to have a higher number of average modification sites *per* transcript (average 2.7), especially YTHDF2 with ~4.7 sites per transcript. Moreover, all four multi-modified YTH transcripts had modification sites within exons or the 3′UTR, suggesting that they are m^6^A modified rather than modified at the cap-adjacent N6,2′‐O‐dimethyladenosine (m^6^A_m_) site [57].

One class of cell surface protein, protocadherins, were commonly found to have a high number of modification sites, exhibiting an average of 4.5, and range of 0–28, modification sites per transcript (Supplementary information Table S1). These proteins mediate trans-synaptic interactions and cell to cell communication and have a suggested role in the generation of specific synaptic connections [58]. In addition, classes of proteins which are traditionally associated with synaptic structure and function were also characterised as multi-modified (Fig. 6F). For example, *CAMK2B*, involved in synaptic spine formation and a classic marker of LTP, had 4.0 modification sites in all tissue types and all glutamate NMDA receptor subunits were found to be modified (Supplementary information Fig. S9). Taken together, these results indicate that: m^6^A binding proteins undergo m^6^A autoregulation; protocadherins transcripts are highly multi- modified; and multi-modification by m^6^A commonly occurs on structural and activity-related synaptic transcripts.

Gene ontology annotation of modified transcripts revealed functional processes most significantly enriched in grey matter related to neuronal and synaptic functions, as well as protein binding, cell junctions and cell adhesion, and post-translational modifications, e.g. phosphorylation and ubiquitination (Fig. 6G, SI Tables S2, S3). White matter tissue indicated different processes were regulated by m^6^A modification. For example, zinc finger protein domains, and processes associated with the nucleus and transcription regulation or myelination and axons (Fig. 6G, SI Table S4, S5). Consistent with previous reports for early developing foetal cortical tissue [59], late-stage foetal brain showed enrichment for transcripts which are associated with nervous system development (SI Table S6). Only one third of significantly enriched process terms were shared across all tissues indicating high variability in which transcripts are modified.

We next examined whether the m^6^A topology along mRNA transcripts revealed enrichment for specific biological processes (SI Tables S8–S12). We found transcripts that were methylated in the 5′ region were enriched for functions relating to neuronal processes and synaptic membranes. Exon-methylated transcripts in grey matter were enriched more for cell-adhesion proteins and cell membranes, whereas in white matter for transcriptional related processes. Transcripts methylated in the 3′ region showed the most functional diversity spanning cellular housekeeping functions such as Golgi membrane function and lipid proteins for grey matter and mitochondria function for white matter. These results together suggest that where a transcript is modified regulates specific processes and pathways which may have consequences for both translational processing and physiological function.

Finally, in grey and white matter tissue, modification of transcripts belonging to the disease classification terms, ‘psychiatric disease’ (*p* > 5*10^−13^, GM; *p* > 1.7 *10^−08^, WM) and ‘neurological disease’ (*p* > 1.5 *10^−6^, GM, *p* > 4.5 *10^−5^, WM) (Fig. 6G,) were enriched. For example, schizophrenia, the most significantly enriched disease in all tissue types, had over 250 different schizophrenia-related transcripts methylated in grey matter (*p* = 6.1*10^−9^), white matter (*p* = 2.5*10^−4^) and foetal brain (*p* = 1.4*10^−4^). Furthermore, transcripts associated with other health conditions such as substance abuse disorders and weight gain were highlighted as m^6^A enriched (SI Table S13). This provides evidence that m^6^A regulation of specific protein pathways could be a significant factor contributing to the expression of brain disorders.

## Discussion

Nucleotide modification as a means to modulate plasticity processes has long been hypothesised [60, 61]. Here we presented new insight into m^6^A methylation processes as a fine-tuning mechanism of translation at pre- and post- synaptic sites and which we propose is an intrinsic component of synaptic tagging and capture. We provided evidence that demethylation by ALKBH5 is an active process during short term plasticity and is involved in active translation at ribosomes local to post-synaptic sites. We also showed that the involvement of effector proteins, YTHDF1, YTHDF3 and ALKBH5 differ between short- and long-term plasticity phases and at synapses during synaptic maturation. As m^6^A binding proteins are currently being assessed as pharmacological targets [62], the temporal changes of these effector proteins need to be considered for drug developments targeting cognitive disorders.

Modification of RNA by N6-methyladenosine is known to influence the cellular fate of mRNA through nuclear processing, transcription/translation initiation, and mRNA transport by RNA binding proteins [63–65]. Cell type, availability of m^6^A effector machinery, and m^6^A-RNAs subcellular spatial localisation are factors likely to determine what biological processes are regulated, where and when. In neurons, modified RNAs are transported from the nucleus to cytoplasmic sites in RNA granules for RNA processing, such as translation repression in P-bodies, or RNA translation at synaptic ribosomes. We propose that demethylation during early plasticity at actively translating synaptic ribosomes, as indicated by our findings, is intrinsic to early tag setting and hence early LTP,- a stage which is postulated to be transcription independent. However, mechanisms which may disrupt RNAs being transported to specific spatial locations such as synaptic compartments, may affect, ‘new’ transcription-dependent, later stages of plasticity (L-LTP). Indeed, depletion of the writer *Mettl3*, reader YTHDC1 and eraser ALKBH5 effector proteins, are reported to cause deficits in nuclear export pathways as well as alterations in the abundance of modified and unmodified mRNAs within the nucleus and cytoplasmic compartment [41, 66, 67]. If export into the cytoplasm was greatly decreased, specific RNAs needed for late-stage plasticity to, for example, sustain a tag over time, or activate tag related pathways, may not be available. We therefore postulate that the result of disruption to both local tag setting by demethylation and nuclear export and transport to already tagged synapses could contribute to synaptic impairment. Indeed, in support of this notion, mutations within genes encoding nuclear mRNA export complex proteins are known to cause forms of syndromic intellectual disabilities [68, 69].

Specific classes of mRNAs including the m^6^A effector proteins were found to be multi-modified suggesting that both the autoregulation of the m^6^A machinery and the spatial locality of the modification along transcripts provide further temporal control over the fate of RNAs. Such highly co-ordinated regulation, may for example, involve m^6^A-modified *YTHDF3-AS1* antisense Lnc transcripts modifying YTHDF reader abundance at synapses during critical stages of development including during synaptic maturation. In addition, as indicated from the m^6^A-seq data, these regulated processes are likely to be influenced by, and to act upon, posttranslational modification pathways, e.g. protein phosphorylation, thereby controlling cascades of orchestrated events fundamental to plasticity [70]. The difference between grey and white matter tissue methylome profiles further demonstrate that the cellular context and local environment are fundamental factors dictating m^6^A regulated pathways. With the emerging evidence of white matter myelination plasticity, i.e. activity-dependent formation of myelin contributing to memory consolidation [54, 55], it will be important to determine what factors within myelinating glia environments govern m^6^A-RNA regulatory processes. Future m^6^A/m mRNA studies of cell populations using techniques such as miCLiP-seq [71] to map sites at base resolution, or nanopore technologies [72] to potentially quantify the number of modifications per base, will be valuable in deciphering cell population specific characteristics.

The YTHDF proteins have recently been shown to promote liquid–liquid phase separation and the formation of highly condensed membraneless macromolecular aggregates such as stress granules, P bodies or ribonucleoprotein granules [12, 73, 74]. One recent model proposed [12, 73], suggests that m^6^A multi-modified mRNAs (also termed polymethylated mRNAs) act as a multivalent scaffold for the binding of YTHDF proteins which enhances phase separation into intracellular phase-separated compartments. Given that post synaptic densities (PSDs) and presynaptic active zone regions are recognised to be dynamic molecular assemblies [75, 76] and that evidence suggests that there are nanodomain assemblies such as excitatory synapse PSDs condensates which are distinct from, and do not mix with, inhibitory synapse PSDs condensates [75], we hypothesise that contingent upon the local availability of the YTHDF proteins, and during early phase synaptic plasticity, multi-modified RNA–YTHDF interaction contributes to formation of dynamic PSD and presynaptic nanodomains.

Observations from our study support such a hypothesis;—namely, shortly after synaptic activation we observed an increase of the YTHDF proteins and the ALKBH5 eraser with m^6^A-RNAs at post synaptic sites. Furthermore, multi-modified mRNAs in grey matter tissue were found to be mRNAs encoding proteins involved with PSD synaptic function. The role for the ALKBH5 eraser protein in a putative phase separation process may be to govern the number, or distribution, of m^6^A methylated sites along local mRNAs and hence influence the stability of YTHDF protein interactions. Alternatively, upon synaptic activation, demethylation by ALKBH5 may initiate rapid local protein translation resulting in an increase in local protein concentrations necessary for phase separation to occur. As a consequence, and as previously suggested [75, 77], mutations within single synaptic proteins could modulate, at a network level, synaptic condensed nanodomain assemblies and hence impair synaptic transmission. Examining the involvement of known mutated genes for neurodevelopmental disorders in m^6^A RNA methylation mediated sub-domain assemblies or disassembly of concentrates, e.g. with YTHDF proteins at ePSDs, may provide valuable insight into basic nanophysiology as well as uncover novel protective ‘plasticity’ mechanisms underlying synaptic function.

Our observations that transcripts for cell adhesion proteins such as cadherins which mediate trans-synaptic interactions and cell to cell communication are highly modified, also suggest a role for m^6^A RNA methylation in cell to cell signalling between neurons and between neuronal and non-neuronal cells. Indeed, the transport of modified RNAs between cells may be an additional means by which information is transferred. Such communication processes would have implications for disease and could be a seeding mechanism by which pathology spreads between brain regions in neurodegenerative disorders. How such m^6^A regulated processes contribute to disease pathologies or brain states, such as addiction disorders, that have the potential to be reversed, will provide valuable insight from which new therapies can emerge. However, with growing evidence of significant divergence in plasticity-related protein gene expression between species [78], epitranscriptomic profiling using human brain will be critical for the translation of findings into clinical use.

## Supplementary information

Supplementary Information file

Supplementary material dataset excel file.

Supplementary material video 1.

Supplementary material video 2.

Supplementary material video 3.

Supplementary material video 4.

Supplementary material video 5.

## Data Availability

All data obtained during this study are available from the corresponding author upon reasonable request.
