# Supplementary material for: Modifying the m6A brain methylome by ALKBH5-mediated demethylation: a new contender for synaptic tagging
Source: Mol Psychiatry. 2021 Oct 19;26(12):7141–53. doi: 10.1038/s41380-021-01282-z (PMC8872986; doi:10.1038/s41380-021-01282-z)
Supplement: Supplementary file 1 — Supplementary Information file [file 41380_2021_1282_MOESM1_ESM.docx]

Braulio Martinez De La Cruz (PhD)^1^, Robert Markus (PhD)^2^, Sunir Malla (PhD)^3^, Maria Isabel Haig (BSc)^1^, Chris Gell (PhD)^2^, Fei Sang (PhD)^3^, Eleanor Bellows (PhD)^1^, Mahmoud Awad Sherif (MD)^1^, Denise McLean (BSc)^2,^, Anbarasu Lourdusamy(PhD)^4^, Tim Self (PhD)^2^, Zsuzsanna Bodi(PhD)^5^, Stuart Smith (FRCS)^4^, Michael Fay (PhD)^6^, Ian A Macdonald (PhD)^7^, Rupert Fray (PhD)^5^, Helen Miranda Knight (PhD)^1^*

*helen.knight@nottingham.ac.uk

The supplementary information file includes details of tables within the supplementary material excel file; legends for the supplementary material video files; and 9 supplementary Figures and 1 supplementary Table. The 9 SI figures and 1 SI Table are:

**Figure S1.** Correlation between confocal imaging fluorescent signals generated by antibodies raised against the m^6^A modification.

**Figure S2.** Confocal imaging establishes that m^6^A colocalises with pre-synaptic markers in differentiated neuronal cell lines.

**Figure S3.** Images of 3D SIM data reveal true protein-m^6^A interactions and observations of false signals of overlap. Images were rotated on the x-axis.

**Figure S4.** Colocalisation between m^6^A-RNAs and RNA binding proteins at post-synaptic sites in differentiated SH-SY5Y cells before and 15 minutes after treatment with NMDA.

**Figure S5.** Mean Pearson’s Correlation Coefficients calculated for confocal imaging colocalisation between m^6^A modified transcripts and m^6^A-binding proteins at post-synaptic pre-synaptic sites (dTE671 cells) and pre-synaptic sites following activation with NMDA and KCl (dSH-SY5Y and dTE671 cells).

**Figure S6.** Fluorescent signal intensity of a puromycin-labelled channel in treated non-activated cells vs treated NMDA-activated cells. Puromycin immunofluorescence in differentiated TE671 cells did not show a significant difference (unpaired *t*-test, p = 0.48) in randomly selected images (*n* = 20).

**Figure S7.** Confocal imaging indicates colocalisation between m^6^A modified transcripts and m^6^A complex proteins at post-synaptic sites before and 15 minutes after NMDA activation and selection for active ribosomes.

**Figure S8.** Expression patterns of *YTHDF1*, *YTHDF3*, *YTHDF2* and *ALKBH5* in the human developing brain.

**Table S1.** m^6^A modified protocadherins transcripts which are found multi-modified in grey and white matter and foetal brain tissue.

**Figure S9.** Coverage plots from m^6^A-seq showing representative examples of multi-modified site transcripts in grey matter tissue (*GRIN2A* and *YTHDF1*) and in white matter tissue (*CAMK2b* and *YTHDF2*).

**Supplementary material excel file.** GO analysis tables of m^6^A-seq of grey and white matter and foetal brain tissue. These include:

Table S2. Gene Ontology functional annotation of m^6^A modified transcripts in parahippocampal adult grey matter.

Table S3. Gene ontology functional annotation of m^6^A modified transcripts in parahippocampal grey matter listed by fold enrichment.

Table S4. Gene ontology functional annotation of m^6^A modified transcripts in parahippocampal white matter.

Table S5. Gene ontology functional annotation of m^6^A modified transcripts in parahippocampal white matter listed by fold enrichment.

Table S6. Gene Ontology functional annotation of m^6^A modified transcripts in foetal 22-30 weeks whole brain.

Table S7. Gene ontology functional annotation of 5’ region m^6^A modified RNAs modified in adult parahippocampal grey matter.

Table S8. Gene ontology functional annotation of exonic m^6^A modified RNAs modified in adult parahippocampal grey matter.

Table S9. Gene ontology functional annotation of m^6^A-RNAs modified within the 3’ regions in grey matter in the adult parahippocampus.

Table S10. Gene ontology functional annotation of 5’region m^6^A modified RNAs modified in adult parahippocampal white matter.

Table S11. Gene ontology functional annotation of exonic m^6^A modified RNAs modified in adult parahippocampal white matter.

Table S12. Gene ontology functional annotation of m^6^A-RNAs modified within the 3’ regions in white matter in the adult parahippocampus.

Table S13. Disease classifications indicated to be enriched for m^6^A modified transcripts in adult human parahippocampal grey and white matter and whole foetal brain.

**Supplementary material video files:**

**Supplementary material video 1.** Cellular response to KCl application by Fluo4-AM calcium imaging in dTE671 cells. Images were captured over a 90 second timeframe on a LSM710 confocal microscope.

**Supplementary material video 2.** Cellular response to NMDA application by Fluo4-AM calcium imaging in dTE671 cells. Images were captured over a 90 second timeframe on a LSM710 confocal microscope.

**Supplementary material video 3.** Video of 3D super resolution structured illumination microscopy images of differentiated human neuronal SH-SY5Y neuronal processes immunolabelled with anti-m^6^A and anti-YTHDF1.  Zoomed in regions show sites of colocalisation between m^6^A modified RNAs and the reader protein YTHDF1 in 3D space. SIM resolution limit, 120-140nm XY dimension and 250nm in Z dimension.

**Supplementary material video 4.** Video of Scanning TEM of m^6^A modified RNAs at synapses in the CA3/4 region of the mouse hippocampus. Images were generated on a JEOL 2100F TEM and videos generated by STEM tilting involving a tilt series acquisition at five degree steps.

**Supplementary material video 5.** Video of Scanning TEM of the YTHDF1 reader protein at synapses in the CA3/4 region of the mouse hippocampus. Images were generated on a JEOL 2100F TEM and videos generated by STEM tilting involving a tilt series acquisition at five degree steps.


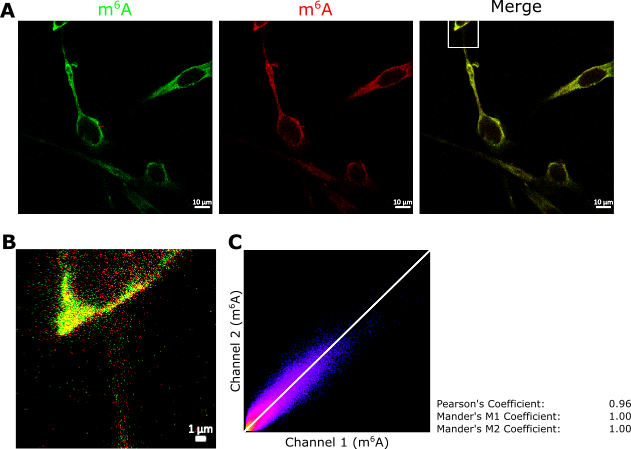


**Figure S1.** Correlation between confocal imaging fluorescent signals generated by antibodies raised against the m^6^A modification. A mouse monoclonal antibody obtained from Merck millipore (MABE 1006, clone 17-3-4-1), a rabbit monoclonal antibody from Abcam (ab190886) were compared for colocalisation using confocal microscopy in SH-SY5Y cells. **A)** Visual comparison of the Abcam (ab190886) and MABE 1006, clone 17-3-4-1 antibodies indicates colocalisation of signals depicted as yellow. *n* = 5. **B)** Inset from (A) indicates high overlap shown as yellow. **C)** 2D intensity histogram shows high colocalisation and similar fluorescence intensity between the two antibodies. The Mander’s M1 and M2 coefficients assesses overlap of one channel only at regions covered by the other channel and indicated a perfect degree of colocalisation.

**
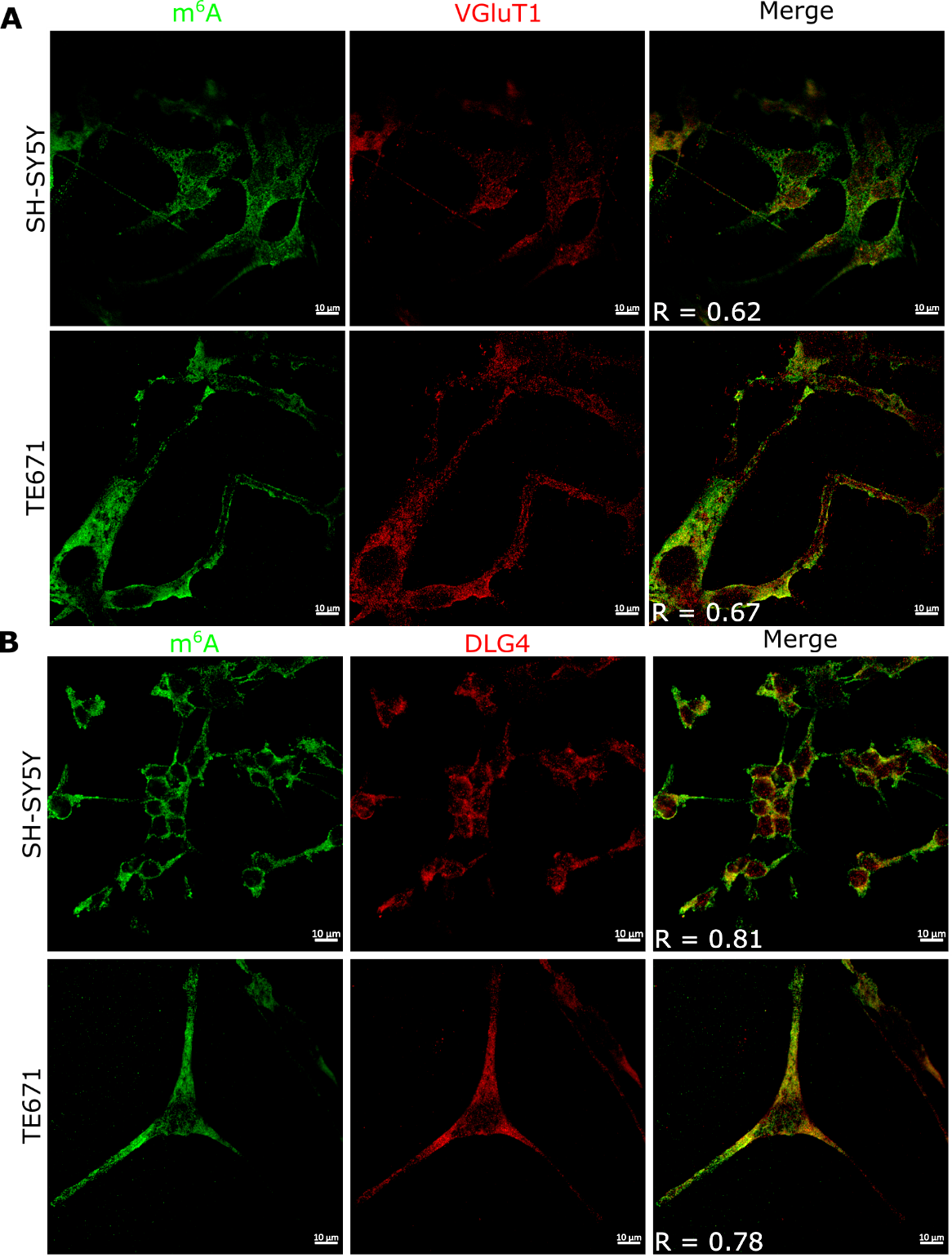
**

**Figure S2.** Confocal imaging establishes that m^6^A colocalises with pre-synaptic markers in differentiated neuronal cell lines. **A)** m^6^A and pre-synaptic marker VGluT1 and **B)** the post-synaptic marker DLG4 [PSD-95], show some colocalization (VGluT1, PCC R ~0.64; DLG4, PCC R ~ 0.80 ) especially in processes (*n* = 10 ). Colocalization was quantified for the whole field of view.

**
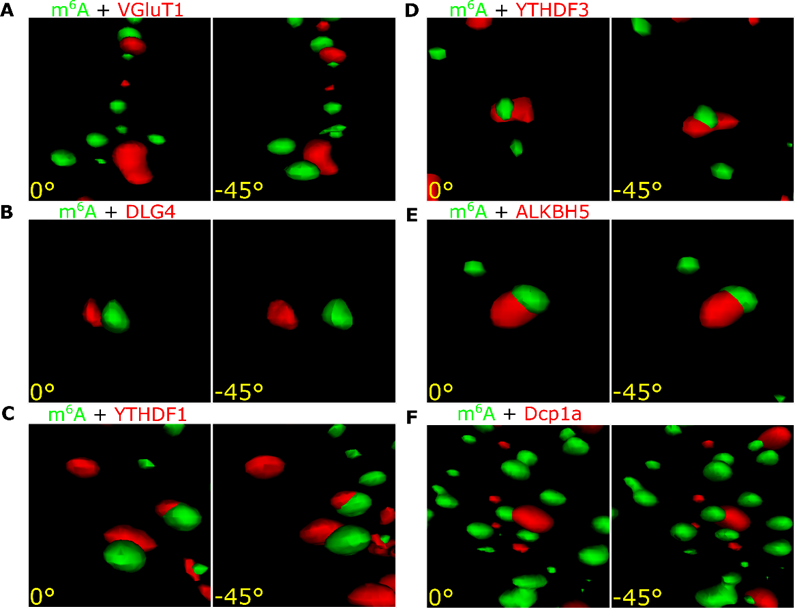
**

**Figure S3.** Images of 3D SIM data reveal true protein-m^6^A interactions and observations of false signals of overlap. Images were rotated on the x-axis. **A-B)** Some infrequent instances of colocalisation between m^6^A and pre- and post- synaptic markers in a 2D image were found to be false positives when rotated as a gap of approximately 120 nm became evident. **C-E)** m^6^A (green) readers YTHDF1, YTHDF3, the eraser ALKBH5 (red) were found always to be directly adjoining with one m^6^A modification signal providing evidence of direct interaction. **F**) Dcp1a (red) was also found to be always directly adjoin m^6^A modified transcripts but commonly one Dcp1a molecule was observed between two m^6^A modification signals.


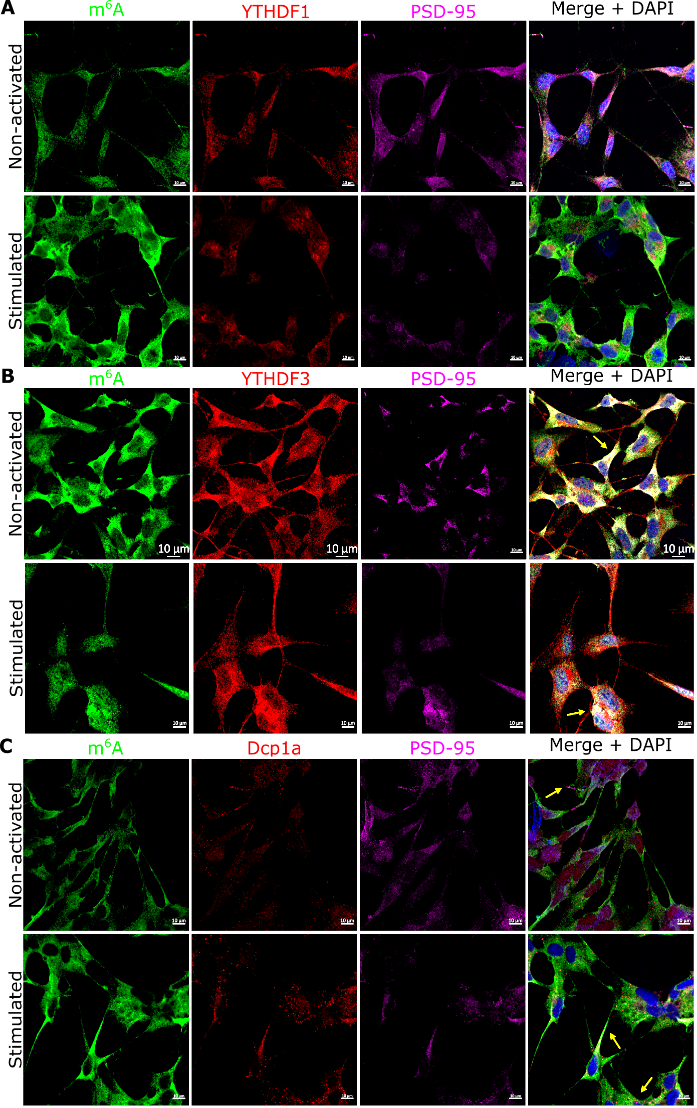


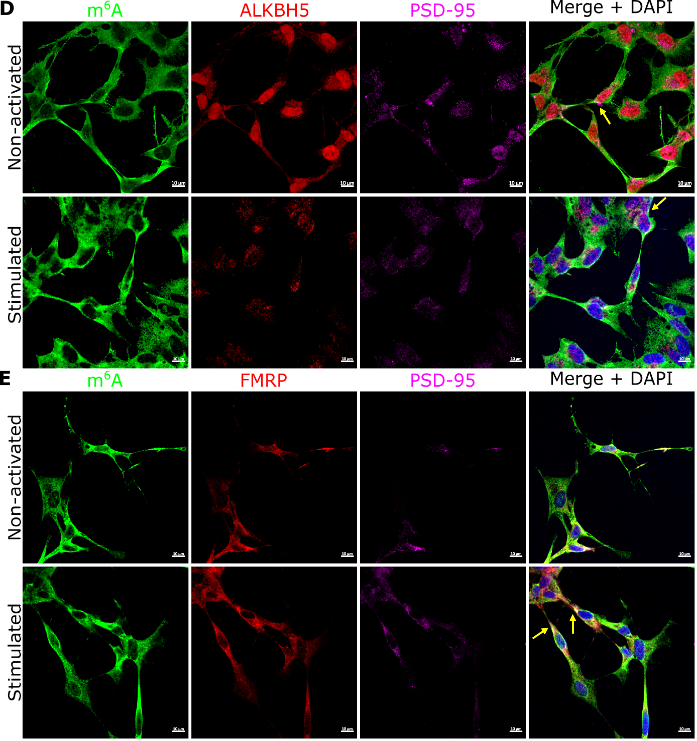


**Figure S4.** Colocalisation between m^6^A and RNA binding proteins at post-synaptic sites in differentiated SH-SY5Y cells before and 15 minutes after treatment with NMDA. **A-D)** Overlap between m^6^A-modified RNAs and m^6^A readers proteins (YTHDF1 and YTHDF3), the eraser protein (ALKBH5), and a P body marker (Dcp1a) at post synaptic sites. **E)** FMR1/FMRP colocalisation with m^6^A-modified RNAs increased following activation, as shown by white and light pink colouring around the cytoplasm and neuronal processes. All confocal microscopy experiments were repeated twice and at least ten images were capture each time, resulting in approximately 700 confocal images captured.


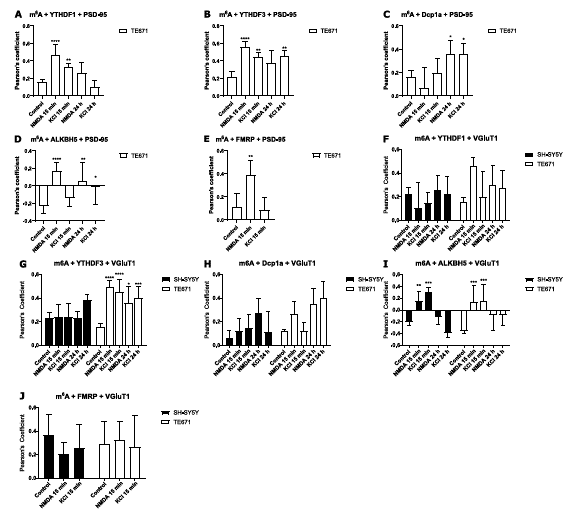


**Figure S5.** Mean Pearson’s Correlation Coefficients calculated for confocal imaging colocalisation between m^6^A modified transcripts and m^6^A-binding proteins at post-synaptic pre-synaptic sites (dTE671 cells) and pre-synaptic sites following activation with NMDA and KCl (dSH-SY5Y and dTE671 cells). A significant increase in colocalisation of m^6^A-modified transcripts with YTHDF1 (**A**) YTHDF3 (**B)** at 15 minutes and 24 hours; and with ALKBH5 (**D)** and FMRP (**E**) at 15 minutes was observed in post-synaptic sites. An increase of m^6^A- RNAs with Dcp1a at post synaptic areas was evident only at 24 hours. Colocalisation of m^6^A-modified transcripts with YTHDF1 **(F)**, Dcp1a **(H)** or FMRP **(J)** did not significantly change at presynaptic sites. However, similar to post-synaptic regions, a significant increase in colocalisation of m^6^A-modified transcripts with YTHDF3 (**G)** and with ALKBH5 (**I)** was observed in pre-synaptic areas after 15 minutes. Error bars denote 95% CI. * p ≤ 0.05, ** p ≤ 0.005, *** p ≤ 0.0005, **** p ≤ 0.00005. All experiments were repeated twice and >10 images were capture each time resulting in approximately 700 confocal images captured.

**
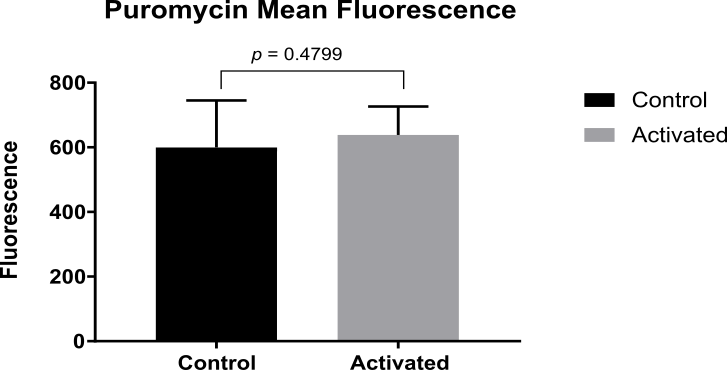
**

**Figure S6.** Fluorescent signal intensity of a puromycin-labelled channel in treated non-activated cells vs treated NMDA-activated cells. Puromycin immunofluorescence in differentiated TE671 cells did not show a significant difference (unpaired *t*-test, p = 0.48) in randomly selected images (*n* = 20).

**
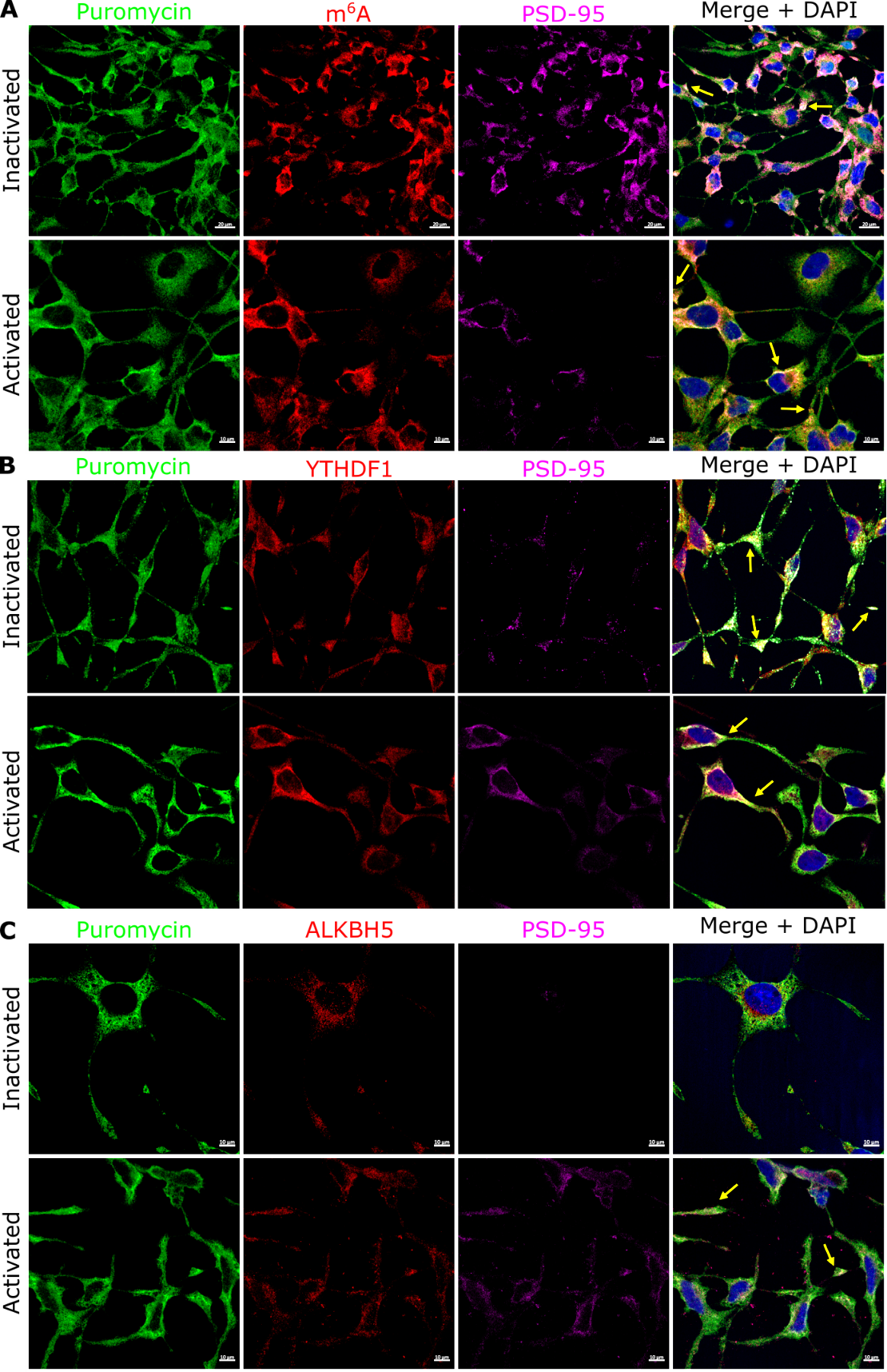
**

**
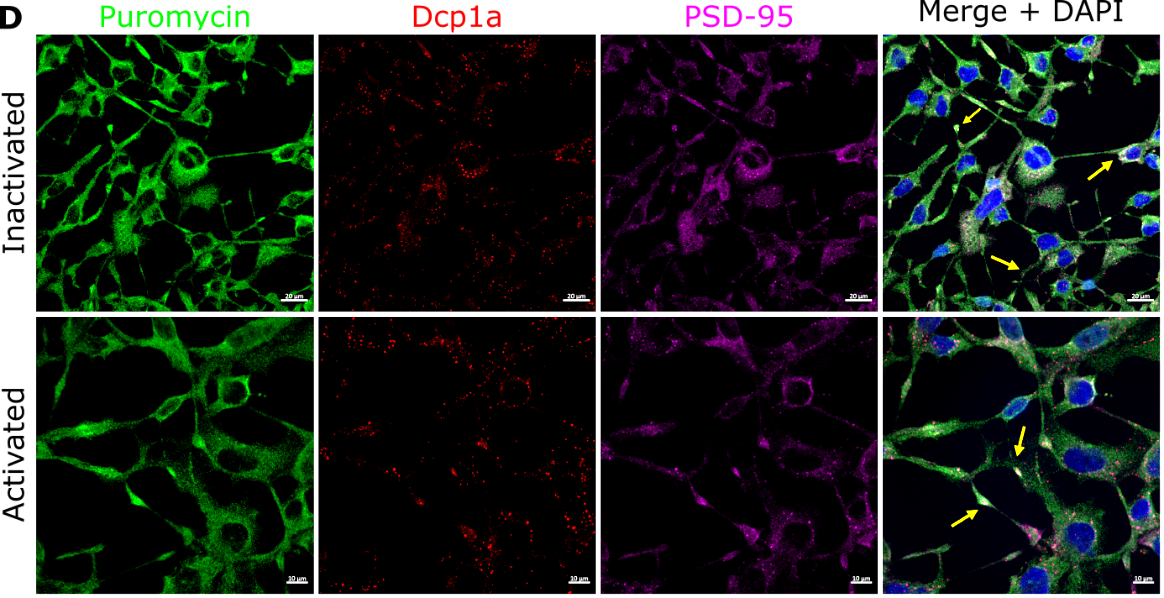
**

**Figure S7.** Confocal imaging indicates colocalisation between m^6^A modified transcripts and m^6^A complex proteins at post-synaptic sites before and 15 minutes after NMDA activation and selection for active ribosomes. Overlap in signal between active ribosomes indicated by the puromycin signal in green and **A)** m^6^A-modified RNAs, **B)** YTHDF1, **C)** ALKBH5, and **D)** Dcp1a, at post synaptic sites. All experiments were repeated twice and >10 images were captured per experiment.


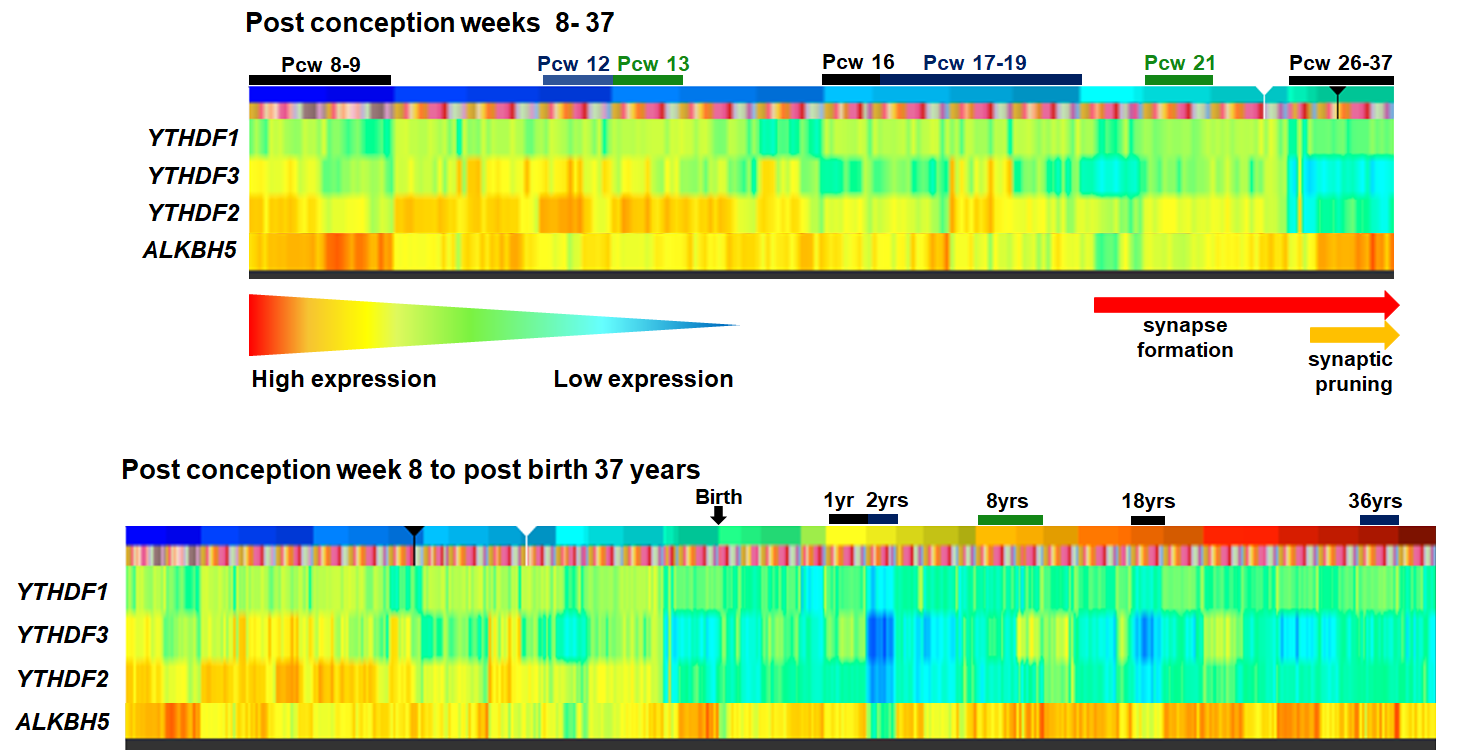


**Figure S8.** Expression patterns of *YTHDF1*, *YTHDF3*, *YTHDF2* and *ALKBH5* in the human developing brain. Top, RNA-seq expression data for *YTHDF1*, *YTHDF3*, *YTHDF2* and *ALKBH5* genes generated for the age developmental period 8-37 pcw across 16 brain regions*. Periods during which synaptic maturation and synaptic pruning occur (pcw 20-37) are highlighted by red and yellow arrows. During early synapse formation, *YTHDF3* expression is moderate but decreases in a later stage during which synaptic pruning occurs. In contrast, *YTHDF1* and *ALKBH5* both show an overall increase in expression during the later stages of synaptic formation and remain relatively high during the period of synaptic pruning. Bottom, RNA-seq expression data for *YTHDF1*, *YTHDF3*, *YTHDF2* and *ALKBH5* genes generated for 8-37 pcw, postnatal and into adulthood 0-40 years across 16 brain regions. Time of birth is indicated by a black arrow. *YTHDF1* and *AlKBH5* show similar spatio-temporal patterns of expression over time up to mid adulthood although *ALKBH5* has overall higher expression than *YTHDF1* and their spatio-temporal patterns differ from YTHDF2 and YTHDF3. Data from the BrainSpan Atlas of the Developing Human Brain^45^ database and figure adapted using the © 2010 BrainSpan Atlas of the Developing Human Brain. Available from: <https://www.brainspan.org/>.

*Regions examined were the: Prefrontal cortex; ventrolateral/medial and orbital frontal cortex; Primary motor cortex, primary somatosensory cortex, posterior inferior parietal cortex; primary auditory temporal cortex, posterior superior temporal cortex, inferior temporal cortex, primary visual cortex, hippocampus, amygdala, striatum, mediodorstal nucleus of the thalamus, cerebellar cortex.

**Table S1.** m^6^A modified protocadherins transcripts which are found multi-modified in grey and white matter and foetal brain tissue. Mean denotes the average number of modifications across a single transcript per tissue.


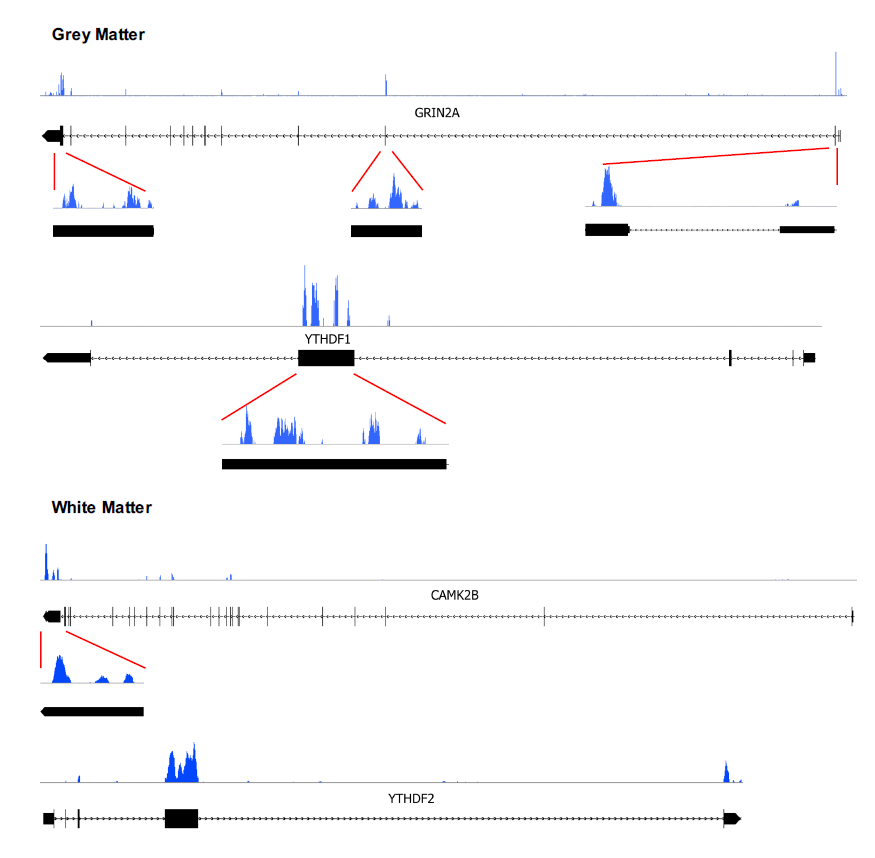


**Figure S9.** Coverage plots from m^6^A-seq showing representative examples of multi-modified site transcripts in grey matter tissue (*GRIN2A* and *YTHDF1*) and in white matter tissue (*CAMK2b* and *YTHDF2*). Peaks represent the mean peak coverage of replicates subtracted by the input peak coverage.
